# An interview with David Normando

**DOI:** 10.1590/2177-6709.24.2.032-039.int

**Published:** 2019

**Authors:** David Normando, Carlos Flores-Mir, Jorge Faber, Leopoldino Capelozza, Weber Ursi

**Affiliations:** 1 » Associate Professor, Federal University of Pará, School of Dentistry, Belém, Brazil. » Coordinator, Brazilian Association of Odontology - Pará Chapter, Graduate Program in Orthodontics, Belém, Brazil. » Editor Emeritus, Dental Press Journal of Orthodontics. » Associate Editor, Progress in Orthodontics.; 2 » Professor, University of Alberta, Department of Dentistry, Division of Orthodontics (Edmonton, Canada).; 3 » Former Chief-Editor, Journal of the World Federation of Orthodontists and Dental Press Journal of Orthodontics. » Researcher, Brasília University, Graduate Program in Dentistry (Brasília, Brazil). » Private practice (Brasília, Brazil).; 4 » Private practice (Bauru, Brazil). » Coordinator, Society for the Social Promotion of Patients with Clef Lip and Palate (PROFIS), Graduate Specialization Course in Orthodontics, (Bauru, Brazil). » Professor, Orthodontics Program, Professional Master’s in Dentistry, Sagrado Coração University (Bauru, Brazil).; 5 » Master’s Degree and Doctorate in Orthodontics, School of Dentistry of Bauru, São Paulo University, (Bauru, Brazil). » Full Professor , School of Dentistry, São Paulo State University (São José dos Campos, Brazil). » Editor Emeritus, Revista Clínica de Ortodontia, Dental Press.

**Figure:**
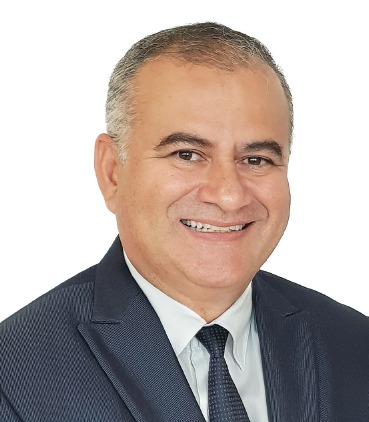

There are several yardsticks to gauge a man’s worth. One of them is, undoubtedly, the respect he earns from his peers. As I organized the questions to be sent to Professor David Normando, made by some of the greatest thinkers in the field of contemporary Orthodontics, I saw the reflex of such respect on the profound inquiring, to which so few would be capable of responding with so much propriety. He has also proven that the stars that shine brightest in the celestial sphere are not restrict to the borders of certain countries or regions. They may become references to us all, wherever they are or come from. However, I must confess that I feel especially proud to know that this icon of Orthodontics had to follow a hard and tortuous path, like the bayou. In the public schools where he studied, in remote cities of the Brazilian Amazon region, his education in Orthodontics required bus travels between Belém and Bauru, which together added up to several journeys around the Earth. On that note, I leave you with a few brief snapshots of his fascinating life history. David has a unifying personality that brings friends closer and organizes teamwork with clockwork precision. This quality has greatly contributed to the growth of scientific production in Dentistry in the Northern Region of Brazil, which culminated in the establishment of the first Doctorate Program in Dentistry in the region. He also has the privilege to have Thiene by his side, his wife and confidant, with whom he had two children, Gabriel and Matheus. A beautiful family that professes the ethics of hard and honest work and happiness.

Jorge Faber (interview coordinator)

Há várias métricas para se aquilatar um homem. Uma delas é, sem dúvida, o respeito que instila em seus pares. Ao organizar o conjunto de perguntas direcionadas ao Prof. David Normando, feitas por alguns dos maiores pensadores da Ortodontia contemporânea, vi o reflexo desse respeito em questionamentos profundos que poucos poderiam responder com tanta propriedade. 

Ele também demonstra que as estrelas mais brilhantes na esfera celeste não estão restritas às delimitações de países ou regiões e se tornam referência para todos, onde quer que estejam e de onde quer que venham. Entretanto, confesso, sinto um orgulho especial por saber que esse ícone da Ortodontia trilhou um caminho tortuoso como os igarapés nas escolas públicas onde estudou, em cidades remotas da Amazônia brasileira; sua formação ortodôntica requereu viagens de ônibus entre Belém e Bauru que perfazem voltas ao redor da Terra. Nisso, cito aqui pequenos flashes de sua bonita história de vida. David tem uma personalidade agregadora que aproxima os amigos e organiza com precisão estatística as equipes de trabalho. Essa virtude muito contribuiu para o crescimento da produção científica da Região Norte do Brasil na Odontologia, culminando na criação do primeiro curso de doutorado em Odontologia nessa região. Ele ainda tem o privilégio de ter ao seu lado Thiene, sua esposa e confidente, com quem tem dois filhos, Gabriel e Matheus. Uma linda família que professa os valores da ética, do trabalho árduo e honesto, e da felicidade.

Jorge Faber (coordenador da entrevista)

1) Professor David Normando, I was very happy to be invited to be part of this distinguished team of professionals that are responsible for asking questions to one of the most qualified Brazilian orthodontists, both clinically and, mainly, in terms of scientific methodology and as an editor of scientific journals. Therefore, I would like to know: which of these professional dimensions motivates you the most currently and at the professional stage in which you find yourself today? (Weber Ursi)

My dear friend, thanks for your affection and admiration, which are mutual. 

I have the impression that, after the 40^th^ year of life, we need to have other activities. The learning curve of the chosen profession slows down, and the brain needs new challenges and learnings. I currently read a lot about Biostatistics, with as much pleasure as about Orthodontics. I like everything I do, but cannot see myself doing a single activity for 40 hours in a week: however, science is my best “sport”. I consider the position of editor as an exercise of citizenship. Together with reviewers, we play a fundamental role in screening what is most reliable to reach the eyes of our readers - most of them general dentists -, and then be passed on to our patients. This role is fundamental for our clinical activity and, therefore, for our society.

2) How do you define successful stability? Are the ideal goals of tooth position stability justifiable? Our understanding of the continuous facial changes along adult life has improved immensely. How do we connect this new understanding to the concept of occlusal stability? (Carlos Flores-Mir)

I am convinced that our stability definitions are very different from those of our patients’ opinion. When a patient comes to my office for a post-treatment control visit, I always repeat the same question: “How is life, and how are your teeth?” I learned this approach from Professor Omar Gabriel. If the answer is “Everything is fine”, I will evaluate occlusal stability from the perspective that whatever I see does not bother the patient. It is important, obviously, to inform the patient about any changes; but we should give them information about whether any retreatment is necessary. Unfortunately, if you let this information be provided to them by another specialist, you will be held hostage to that colleague’s good professional conduct and ethics. 

3) In my opinion, based on the literature and on extensive clinical and teaching experience, post-treatment problems have already been defined a long time ago. According to the perspective that classifies post-treatment changes into relapse and instability, the orthodontist should be responsible for sharing this information with the main stakeholder - the patients or their guardians -, and should adopt mutually agreed procedures, in the understanding that the treatment is not over when the appliance is removed. Numerous orthodontists are reluctant to accept these potential post-treatment changes associated with growth pattern, maturation and ageing, all already inexorably defined by science. They counter this understanding with the possibilities of absolute stability, which may happen, but cannot be prognosticated under any known method. In this context, Nanda and Burstone^1^ stated that we believe that stability is not a problem, and generations of orthodontists have continuously been educated to believe in this concept. What is your position about this process, and what should be the conceptual, teaching and clinical approaches derived from it? (Leopoldino Capelozza)

Again, this is a popular concept in Brazil. In respected scientific events, I have never heard anyone challenging orthodontic treatment instability and physiological ageing of occlusion. I see an opportunistic trait in the concept that excellent occlusion is immutable. The body is not immutable. Numerous studies have reported that even cases that were completed with excellent results may have instabilities. Even though these studies may have methodological flaws, we should base our understanding in this scientific premise. If any colleagues reading this interview believe that their cases will be stable without a retainer, I invite them to conduct an investigation in which we would follow up consecutive clinical cases that did not use retention. I will be the first to spread the word that orthodontic science, as well as orthodontists in the same league of luminaries such as Burstone, Little, Nanda and Capelozza, were wrong. For now, I prefer to keep retentions.

4) Could you please list four studies in the last ten years that have significantly changed Orthodontics for you? The underlying idea here is that little of what has been published ends up decanting in our clinical orthodontic practice, and many of us continue to use the concepts learned in courses with “experts”, rather than concepts based on scientific evidence. (Weber Ursi)

I believe I had an excellent orthodontic education, in which clinical cases that illustrated treatment protocols were accompanied by presentations of scientific studies that justified the decisions made. Obviously, we do not have evidence for all our questions, and the verb “to doubt” should always be present in the mind of any researcher, as we work to ensure that it is also in the mind of the general dentist. This interview may not raise a lot of interest, because it has more reference citations than clinical cases. If we, researchers, keep fighting this approach, we will be talking just to ourselves until the day we die. This does not mean that there is incoherence between the act of showing clinical cases and the science. This was one of the first lessons I learned, as I mentioned in the beginning of this paragraph. 

Now, regarding the choice of studies, the answer is not that simple. Forgive me for the lack of modesty, but one of the most important publications I have seen in Orthodontics was written by me, under the supervision of Professors Capelozza and Omar Gabriel. Conducted when I was a resident at the *Hospital de Reabilitação de Anomalias Craniofaciais / Universidade de São Paulo* (HRAC-USP), this study^2^ found evidence that the main effect of changes in the face of individuals with unilateral cleft lip and palate were a result of the lip, and not of the palate, surgery, as was believed before. And, lo and behold, despite this conclusion, ratified by a systematic review and meta-analysis,^3^ the lip surgery has kept, until today, practically the same surgical protocol in terms of intervention time. Einstein believed that, *“It is easier to smash an atom than a prejudice”.*

The difficulty in choosing studies that have an impact on our daily clinical routine is focused on the fact that, most times, science ratifies some of the protocols we already use. Therefore, science seems to lose part of its novelty. In science, we call this “confirmation bias”. That is, we believe more in what confirms our beliefs than in what challenges or denies it. Many do not know the difference between technology and science. Maybe the role of orthodontic science is to control quality, turning down technologies that do not have the efficiency advertised by the industry. One good example in this area is the studies that suggested that self-ligating brackets are not more efficient than the conventional ones, when comparing treatment time.^4^

However, it is obvious that there is novelty in science, as the study headed by Professor Hugo De Clerck,^5^ which confirmed the beneficial effects of skeletal anchorage in the difficult mission of orthopedic gain in the treatment of patients with maxillary deficiency. Another beautiful study, conducted by Camila Massaro and Felícia Miranda, under the supervision of Professor Daniela Garib and published in AJODO,^6^ describes the changes in the face and in dentition that accompany ageing. Poetically, the sentence “occlusion is the most stable characteristic of the face along maturation” should lead us into a profound reflection about the way we handle orthodontic treatment prognosis. I understand your question and, in fact, there are some studies that do not have an immediate clinical application, and others whose real importance remains to be perceived.

5) Your activity in graduate education and as editor of the Dental Press Journal of Orthodontics in the last years indicates that you have been an artisan and witness of the rise of Brazilian Orthodontics as a science. From this privileged point of view, which actions do you believe have been carried out to make this possible, and what should be done to keep it progressing? (Leopoldino Capelozza)

I believe that the rise of Orthodontics as a science is the reflex of a public policy for Brazilian graduate studies, also seen in other fields of study. It has been more evident in Dentistry because we have a brilliant generation of Brazilian dentists doing science. In the other fields of science in Brazil, we also have researchers and conference speakers that are among the best in the world, when compared with other countries. The impact of the dental science produced in Brazil is one of the greatest in the world. In contrast, I have a feeling that we will not have thinkers in Orthodontics or Dentistry like you in the future generations. Fortunately, our generation is still active. We have promising young dentists; however, although I see that they have a great ability to do things, I do not see, with a few exceptions, the depth of thought that past generations had. Science requires hard work, but cannot be done without critical thinking, creative originality and resources. Therefore, I am not optimistic about its growth, nor even about maintaining the level of orthodontic science in our country. Not in the short term.

6) What is your opinion about the fact that official Research Funding Agencies seem to assign great importance to the number of studies published by an author, often at the expense of quality and greater care in writing papers? Under all this pressure, don’t we end up with a single object of study divided into several publications, with very few differences between them? (Weber Ursi)

No doubt. In Brazil, because of the mechanisms of research funding, a researcher is practically forced to mentor doctorate, master’s and undergraduate students, in scientific initiation programs. With so much exhalation, there is no time left for inhalation - and science requires it. But I believe that this view will be outdated very soon. This was a necessary step justified by the inefficiency of Brazilian intellectual production. The current area coordination of the Coordinating Agency for Advanced Training of Higher Education Personnel (CAPES) and of the National Council for Scientific and Technological Development (CNPq) have already pointed to other directions, in which more importance will be assigned to greater quality of what is produced.

7) There is no doubt that the orthodontic science produced in Brazil has received increasingly greater exposure all over the world. It is, if not the first in production, definitely one of the top three in the world. This is a result of the number of publications and, in some way, of the implicit quality of the peer review process. How does this affect, if so, the quality of orthodontic treatments provided to patients in Brazil? (Carlos Flores-Mir)

First, I should stress my great joy in receiving such intelligent, pointed questions. The answer is reflexive and may not match the level of the questions. In fact, Brazilian orthodontic and dental sciences are among the most productive in the world. At the same time, and in general, we provide Orthodontics of lesser quality to our population. In general, I repeat. We definitely have excellent orthodontists, some of the best in the world, but there is an overwhelming number of colleagues with serious flaws in their education. They are usually those that do not keep up to date; many have never attended a congress after their specialization course. There are several reasons for that. At the same time, there is a total lack of governmental control of professional education in the field of health sciences. A saturated work market and a large number of poorly educated colleagues are the common ingredients of a recipe for failure. 

8) Treatment time is a controversial concept. Should we always aim at perfection when treating patients, regardless of how long it will take? How do you balance these three things: our conceptually perfect treatment goals; patients’ perception of treatment completion; and our duty to offer the best possible care to patients? (Carlos Flores-Mir)

**Figure 1:**
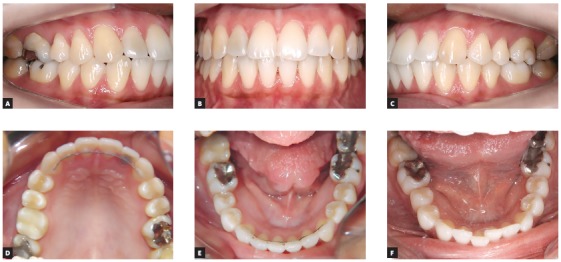
Clinical case in retention for 6 years (A-E), with good outcome. Retention protocol included fixed retention in anterior regions of maxilla and mandible using a 0.021-in 6-filament coaxial archwire. Nine years after treatment, three years after fixed retention was removed, as requested by the patient, mandibular teeth misalignment was found (F).

I try to aim at excellence, but I used to be stricter with my clinical results. One scenario is that in which you aim at a standard of excellence when your patient has 12-14 months of treatment; another is when the patient has already been under treatment for 4-5 years. Every time my patients want to have the appliance removed, I try to explain the risks of an inadequate treatment finishing; but this decision is theirs, whether we like it or not. In these cases, when treatment time has been over the acceptable limit of time and there is still need to complete the treatment of posterior teeth, I adopt the conduct of removing the appliance and waiting for posterior occlusion settling. It tends to occur spontaneously, but not in all cases.^7^ Therefore, I focus on completing the treatment of the anterior region, which has greater esthetic impact and is where the greater post-treatment changes occur. In this region, my protocol includes maxillary and mandibular fixed retention (Fig 1). 

9) You had the unique opportunity of working with indigenous populations relatively isolated from our culture. Does the value of smile esthetics for an individual’s self-esteem among Brazilian indigenous seem to be different from the value we assign to it? (Jorge Faber)

This is a very difficult question to answer. In fact, as all questions in this interview. I have kept contact with semi-isolated communities since the time I lived in an inland area of the Brazilian state of Acre. In *Xingu*, I had a hard time until I finally managed to take photos of some indigenous smiling. Among the people of the *Arara* ethnicity, who lived in the *Laranjal* village, this was an almost impossible mission. Nobody could make them smile, regardless of their occlusal condition, which was predominantly normal. However, my mission was very simple among the indigenous of the same ethnicity living in the *Iriri* village, where there was a prevalence of mandibular prognathism associated with longer faces. Therefore, what I can vouch for after our journeys is the presence of a fantastic cultural diversity. Maybe not more nor less than the diversity we find among ourselves. 

10) The diagnosis of malocclusion and the treatment prognosis continue being the basis for orthodontic practice and for any other action for treating diseases. The classification of malocclusions according to the molar relationship is both efficient to describe this condition and inept to diagnose its cause(s) and, therefore, to define a prognosis for the treatment to be implemented, no matter how good the evidence in the literature. Despite that, this classification reigns supreme, but, to gain specificity and improve prognosis, depends on complementary information that is not always adequate, because of the type of knowledge available. What justifies this resistance, particularly after studies in the literature confirmed the role of facial growth pattern as a primary etiological factor in most malocclusions? (Leopoldino Capelozza)

Confirmation bias, again. We believe in what our parents and teachers taught us, and tend to doubt everything that goes against it. Many believe that any challenge to the concepts received is an offense to their ancestors. What parent would be happy to see a child doubt their teachings? Most maybe not; but those parents that see knowledge as the basis of education would applaud it. I have no doubt, and neither do you, that if Angle, a genius of Orthodontics, were alive, he himself would have already changed his classification. Maybe he would have already eliminated the classifications, in face of so much variation in human occlusion. We, humans, love to classify. Angle’s classification was important in the beginnings of Orthodontics because of its simplicity and capacity for organization at that time, when we were beginning to understand our specialty. Maybe Orthodontics still has room for this classification system, but as long as it is included in a broader process, which should be as complex as occlusal variability and its causes. 

11) How do you see the relation of the orthodontic industry with our specialty? To what extent is it a win-win relationship and at what point does a certain loss of independence, which has always been a characteristic of Orthodontics, begin? (Weber Ursi)

I think some orthodontists depend on the industry, but many remain independent. A study published in 2017 did not find any significant associations between effect direction (treatment outcome) and the declaration of industry sponsorship or conflict of interest.^8^ Orthodontics will be what we are, including the industry. I think it does not make sense to put a fight against the industrial sector in Orthodontics. In some countries, it is the most important source of funds for scientific research. In Brazil, it is the State. The serious problem is the misguided management of the education of Brazilian orthodontists. We become easy preys because of so much disbelief in science. We have reversed the expected order. We have an excessively liberal education, but science management is in the hands of the State. The industry understands, by means of surveys, which way we tend to go, and goes the same way. If we choose the best direction, it will follow us. But if we choose rock bottom, it will build pits for us to hide ourselves.

12) What is your perspective on the near future of Orthodontics, considering this avalanche of new uses for not so new treatments - which is surprising, because these novelties attract unusual attention from professionals in the area, despite the fact that they are extremely complex operationally and require patient collaboration? Are we going to repeat the recent past, and everything will just pass like a fad, or are we really moving in the direction of a new horizon? (Leopoldino Capelozza)

Don’t you think it is intriguing that there is so much resistance against changing basic concepts used in the diagnosis of malocclusions and, at the same time, so much leniency in accepting so many new treatment approaches? Many do not realize it is the diagnostic error that leads us to wrong choices for the most adequate treatment approach. 

It is evident that we currently have more treatment resources, which have produced significant changes in our Orthodontics. The use of skeletal anchorage is probably the best example. But, for 30 years in Orthodontics, I have witnessed numerous treatment methods that just passed like a storm. And it was science that stilled the winds. Many others will come and, while our orthodontists do not have an education focused on the reliability of scientific knowledge, we will be at the mercy of numerous storms. In Brazil, they will certainly not be just drizzles. Of course, after some of us soak to the bones, others will learn how to dodge the rain.

13) The recommendation of prophylactic extraction of third molars remains controversial. Evidence is definitely not categorical. How do you approach this question with your patients? (Carlos Flores-Mir)

**Figure 2:**
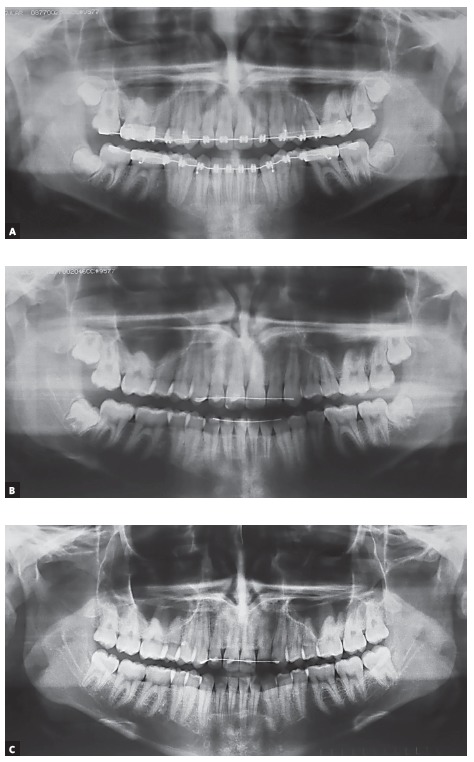
Patient aged 14 years and 6 months old at orthodontic treatment completion (A). When examining this radiograph, 52% of oromaxillofacial surgeons and 37% of orthodontists indicated tooth #48 extraction. Extraction of tooth #38 was indicated by 44% of surgeons and 48% of orthodontists. Two years later, another panoramic radiograph (B) was used to follow up eruption of the third molar at risk of impaction. At age of 19 years and 3 months, after follow-up of almost five years, all third molars had erupted spontaneously (C).

Source: Bastos et al,^9^ 2016.

I am conservative in terms of third molars, except when there are clear signs indicating the need to extract them. In asymptomatic cases, I follow them up practically to exhaustion. I have seen numerous third molars that erupted spontaneously, and that had received a previous “death sentence” (Fig 2). We have demonstrated, in some studies, that we still do not have the ability to reach a relatively reliable definition of which third molar will erupt and which will become impacted.^9^ ^,^ ^10^ Therefore, in asymptomatic cases, follow-up is the most reasonable option.

14) Many suggest that the absence of third molars is an evolutionary stage of the human species. Do you think this claim has merit, and why? (Jorge Faber)

There are many questions about this issue. Several authors assign the increase in the prevalence of third molar agenesis to changes in diets since we started eating more processed foods. However, there are other hypotheses to explain the increase in frequency of third molar agenesis, such as agenesis as a result of an evolutionary process, as opposed to impaction, or agenesis as the result of a genetic mutation. Based on science, I recommend reading a doctorate thesis defended in the Harvard University Department of Human Evolutionary Biology.^11^ Yes, only orthodontists like you, and a few colleagues that might have reached the end of this interview, would be interested in this topic. The thesis is available at https://dash.harvard.edu/handle/1/33493544. 

The author conducted a systematic review and meta-regression using data about agenesis and impaction in modern populations all over the world. To analyze agenesis, 92 studies that included 63,314 individuals were examined. Results indicated that mean prevalence of agenesis in the world is 22.63%. However, while some studies reported a prevalence of only 5.32%, others found a 10 times greater occurrence, of 56%. Part of this diversity is explained by geographical variation. The author examined data about four populations before and after the advent of agriculture, and about two others before, during and after industrialization, and concluded that the hypothesis of natural selection against impaction was the most plausible. In my humble opinion, considering such great variability in prevalence of third molar agenesis in modern human populations, I think that the migratory flows of the species may be associated with this variability. Peter Ungar describes it so well in his book Evolution’s bite, that I received as a gift from a great friend, when he claims that the only mammal that comes closer to human geographical distribution is the brown rat, and this is only because these animals followed us to most places we went to.^12^
